# Patient-derived xenograft models of Fanconi anemia–associated head and neck cancer identify personalized therapeutic strategies

**DOI:** 10.1172/JCI195334

**Published:** 2025-12-16

**Authors:** Jennifer R. Grandis, Hua Li, Benjamin A. Harrison, Andrew L.H. Webster, Joanna Pucilowska, Austin Nguyen, Jinho Lee, Gordon B. Mills, Jovanka Gencel-Augusto, Yan Zeng, Steven R. Long, Mi-Ok Kim, Rex H. Lee, David I. Kutler, Theresa Scognamiglio, Margaret Brandwein-Weber, Mark Urken, Inna Khodos, Elisa de Stanchina, Yu-Chien Lin, Frank X. Donovan, Settara C. Chandrasekharappa, Moonjung Jung, Mathijs A. Sanders, Agata Smogorzewska, Daniel E. Johnson

**Affiliations:** 1 Department of Otolaryngology – Head and Neck Surgery, UCSF, San Francisco, California, USA.; 2 Laboratory of Genome Maintenance, The Rockefeller University, New York, New York, USA.; 3 Immune Monitoring and Omics Lab, Knight Cancer Institute, Oregon Health Sciences University, Portland, Oregon, USA.; 4 Department of Pathology, and; 5 Department of Epidemiology and Biostatistics, UCSF, San Francisco, California, USA.; 6 Department of Otolaryngology – Head and Neck Surgery, and; 7 Department of Pathology, Weill Cornell Medical College, New York, New York, USA.; 8 Department of Pathology, and; 9 Department of Otolaryngology – Head and Neck Surgery, Icahn School of Medicine at Mt. Sinai, New York, New York, USA.; 10 Antitumor Assessment Core, Memorial Sloan Kettering Cancer Center (MSKCC), New York, New York, USA.; 11 National Human Genome Research Institute, NIH, Bethesda, Maryland, USA.; 12 Department of Hematology, Erasmus MC Cancer Institute, Rotterdam, Netherlands.

**Keywords:** Genetics, Oncology, Head and neck cancer

## Abstract

Fanconi anemia (FA) confers a high risk (~700-fold increase) of solid tumor formation, most often head and neck squamous cell carcinoma (HNSCC). FA germline DNA repair defects preclude administration of most chemotherapies, and prior hematopoietic stem cell transplantation limits the use of immunotherapy. Thus, surgery and judicious delivery of radiation offer the only treatment options, with most patients dying from their cancers. A paucity of preclinical models has limited the development of new treatments. Here, we report what to our knowledge are the first patient-derived xenografts (PDXs) of FA-associated HNSCC (FA-HNSCC) and highlight the efficacy of FDA-approved EGFR-targeted therapies in tumors with high EGFR and phosphorylated EGFR levels and the activity of the FDA-approved B-cell lymphoma 2 (Bcl-2) inhibitor venetoclax in a FA-HNSCC PDX overexpressing Bcl-2. These findings support the development of precision medicine approaches for FA-HNSCC.

## Abstract 2

We generated the first patient derived xenograft models of Fanconi anemia-associated head and neck cancer and used these models to identify precision medicine therapies based on genomic, transcriptomic, and proteomic profiles.

## Introduction

Fanconi anemia (FA) is a genomic instability disorder characterized by germline mutations in 1 of 23 genes (*FANCA* to *FANCX*, but most commonly *FANCA*) (1–3), failure to repair DNA interstrand crosslinks, hypersensitivity to DNA-damaging agents, and bone marrow failure (4). Advances in bone marrow transplantation have markedly improved survival rates for patients with FA (5). However, young adults with FA have a dramatically increased risk for developing solid tumors, affecting approximately 75% of those who live to the age of 45 years (6–9). Squamous cell carcinomas (SCCs) of the vulva, esophagus, and head and neck (HNSCC) are most common, particularly HNSCC, which has a roughly 700-fold increased incidence relative to the general population (7, 10, 11). Therapeutic options for FA-associated HNSCC (FA-HNSCC) rely on surgical resection with curative intent due to acute adverse reactions in these patients to DNA-damaging chemotherapeutic agents such as cisplatin (11). Radiation therapy can generally be administered using a graduated volume and dose-escalated approach, although efficacy and treatment-related toxicities remain a concern (11–14). Limited data are available on the use of FDA-approved immune checkpoint inhibitors, although a single case report suggests tolerability in this population (12). To our knowledge, there are no publications reporting a cure of FA-HNSCC if surgery is infeasible. Clinical investigation has been severely hindered by the rarity of this disease and the lack of useful models. Preclinical studies with well-characterized in vivo models of FA-HNSCC are needed to guide the prioritized selection of molecular targeting agents in this rare patient population. We describe the generation of what we believe to be the first patient-derived xenograft (PDX) models of FA-HNSCC. Comprehensive characterization of these PDX models coupled with in vivo therapeutic testing revealed disease heterogeneity and identified potential biomarker-driven therapeutic approaches for this devastating disease.

## Results

### Generation of FA-HNSCC PDX models.

PDX models of FA-HNSCC have not been reported, to our knowledge. We successfully generated 3 independent FA-HNSCC PDX models (FA PDX 1, FA PDX 2, and FA PDX 3) by implanting tumor tissue from 3 patients with FA-HNSCC into the flanks of immunodeficient mice. H&E staining of the PDX models confirmed that all 3 models represented invasive carcinoma (Supplemental Figure 1; supplemental material available online with this article; https://doi.org/10.1172/JCI195334DS1). Moreover, tumor cell morphologies and tumor stromal components were highly similar between early-passage tumors (passages 4, 3, and 7 for FA PDX 1, FA PDX 2, and FA PDX 3, respectively, as shown in Supplemental Figure 1) and late-passage tumors (passages 16, 28, and 23; data not shown) for all 3 models. FA PDX 1 was generated from a stage 2 hypopharyngeal tumor resected from a 13-year-old male individual who died from lung metastases 9 months postoperatively without receiving radiation or systemic therapy. The FA PDX 2 model was generated from a recurrent stage 4 squamous cell carcinoma of the mandible resected from a 38-year-old male individual who also died from the cancer. Prior to that resection, the patient was treated with radiation as well as with paclitaxel and pembrolizumab. FA PDX 3 was generated from an untreated tongue tumor resected from a 27-year-old male individual who experienced a regional metastasis to the neck 9 months after primary resection, was treated with proton therapy, and had not experienced disease recurrence over 3 years. All 3 individuals had germline biallelic *FANCA* variants leading to FA (Table 1). The youngest individual (FA PDX 1) also carried a heterozygous pathogenic germline variant in BReast CAncer gene 2 (*BRCA2*) (also known as *FANCD1*. Like the majority of individuals with FA (15), all 3 patients underwent hematopoietic stem cell transplantation in their first decade of life (for clinical and pathology details, see Table 1, Supplemental Table 1, and the Supplemental Clinical Information).

### Genomic and proteomic characterization of the FA-HNSCC PDX models.

**Figure 1:**
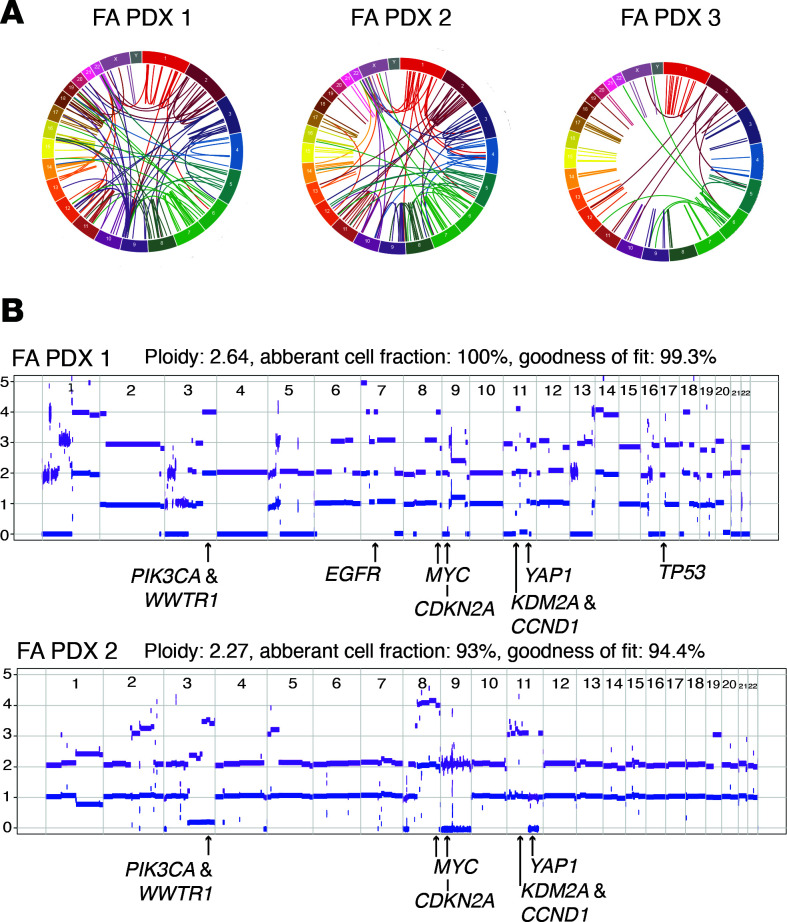
Generation and genomic characterization of FA-HNSCC PDX models. (**A**) Circos plot displaying somatic SV calls in the PDX tumors, with chromosomes indicated on the outer ring. (**B**) Shown are allele-specific copy number plots of FA PDX 1 and FA PDX 2. Blue and purple lines represent minor and total allele counts, with *y* axes indicating relative copy numbers across all chromosomes. The estimated ploidy, tumor purity, and ASCAT model fit for the PDX tumor samples are indicated. Annotated below the graph are key HNSCC oncogenes and tumor suppressor genes with copy number alterations in each sample.

**Figure 2:**
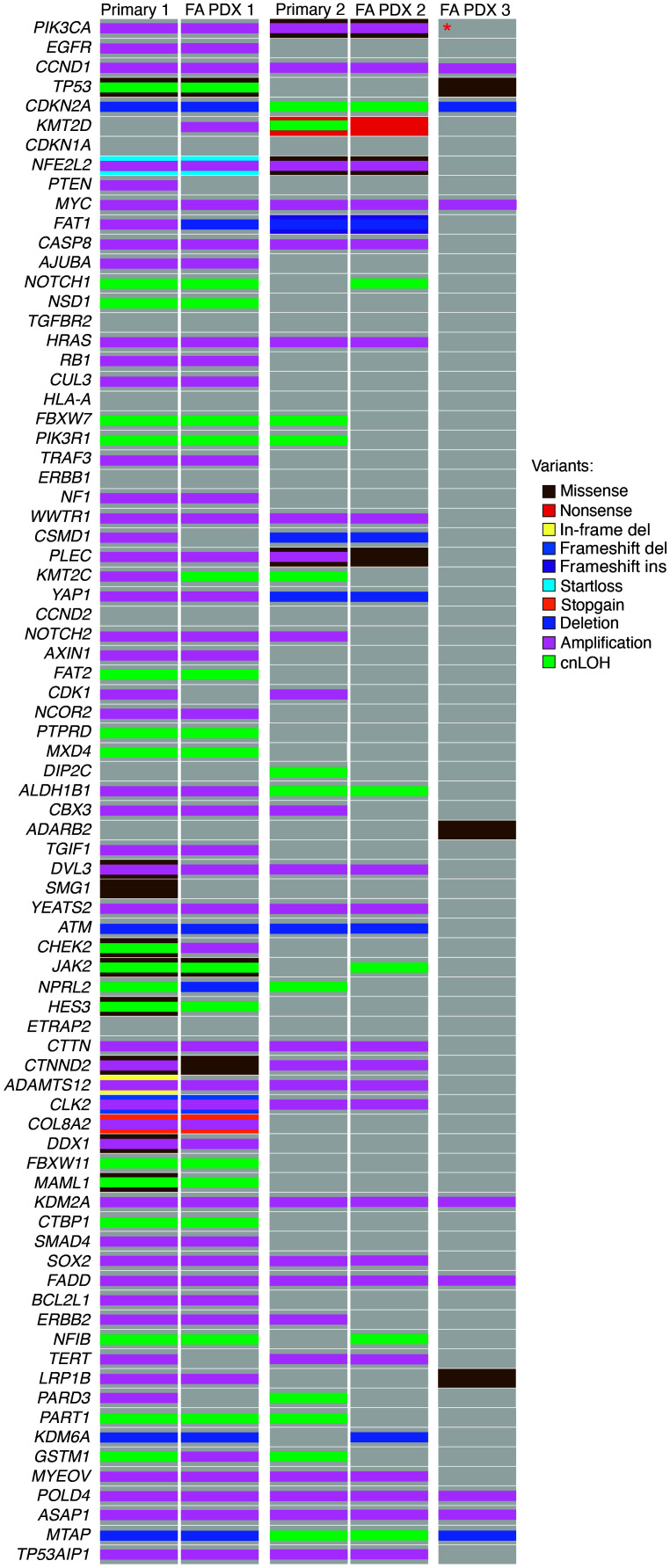
Oncoplot indicating curated HNSCC oncogene and tumor suppressor gene somatic alterations in primary and PDX tumor samples. The key indicates the alteration type by color. The red asterisk indicates that *PIK3CA* had an increased copy number, although the log_2_ fold change of 0.4 did not reach our cutoff of 0.6 to count as an amplification. The primary tissue for FA PDX 3 was not available for analysis. del, deletion; ins, insertion.

Since our FA-HNSCC PDXs represent, to our knowledge, the first such models for this rare disease, we determined the genomic landscape of each model (Figures 1 and 2). All models were derived from patients with germline biallelic *FANCA* variants and were characterized by high numbers of somatic structural variants and somatic copy number changes (Figure 1, A and B, Supplemental Figure 2, and Supplemental Tables 2 and 3), consistent with previous sequencing of FA-HNSCC tumors (16). Among the key alterations noted, FA PDX 1 exhibited a heterozygous germline variant of *BRCA2/FANCD1*, somatic *TP53* pathogenic variants, and somatic gene amplification of *EGFR*, *MYC*, and *PIK3CA*, which encodes the enzyme phosphoinositide 3-kinase (PI3K), as well as *CCND1,* which encodes cyclin D1. The FA PDX 2 tumor model demonstrated a somatic *PIK3CA* missense pathogenic variant and amplification of the *MYC* and *CCND1* genes, among others. FA PDX 3 carried a somatic *TP53* missense variant and amplifications of *CCND1* and *MYC*. Comparison of whole-exome sequencing data revealed a high degree of similarity between early-passage tumors (passages 2, 3, and 2 for FA PDX 1, FA PDX 2, and FA PDX 3, respectively) and late-passage tumors (passages 16, 28, and 23). Single nucleotide variants (SNVs) in early- and late-passage tumors were 96%, 98%, and 97% identical for FA PDX 1, FA PDX 2, and FA PDX 3, respectively. For insertions/deletions (InDels), early- and late-passage tumors exhibited greater than 99% similarity for all 3 models.

We also sought to determine whether the genomic landscape of the FA-HNSCC PDX tumors closely reflected the genomics of the primary tumor. Comparison of somatic variants revealed similar patterns of somatic changes, especially in the most commonly affected genes (Figure 2, Supplemental Figure 2, and Supplemental Tables 2 and 3). As found in other cancers, the PDXs had more structural variants than did the primary tumors (17). In addition, loss of heterozygosity in the *FANCA* locus and the *BRCA2/FANCD1* locus were noted in FA PDX 1 (Supplemental Figure 3). The more deleterious germline *FANCA* deletion variant (del exon 1–6), already present at an allelic frequency below 0.5 in the primary tumor, was replaced by the less deleterious germline splice variant allele (*FANCA* c.1359+1G>C). Similarly, the deleterious *BRCA2/FANCD1* germline variant (*BRCA2/FANCD1* c. 5722-5723del) had a higher allele frequency in the PDX than in the primary tumor, with the PDX most likely representing a purer population of tumor cells than the primary sample that was sequenced.

**Figure 3:**
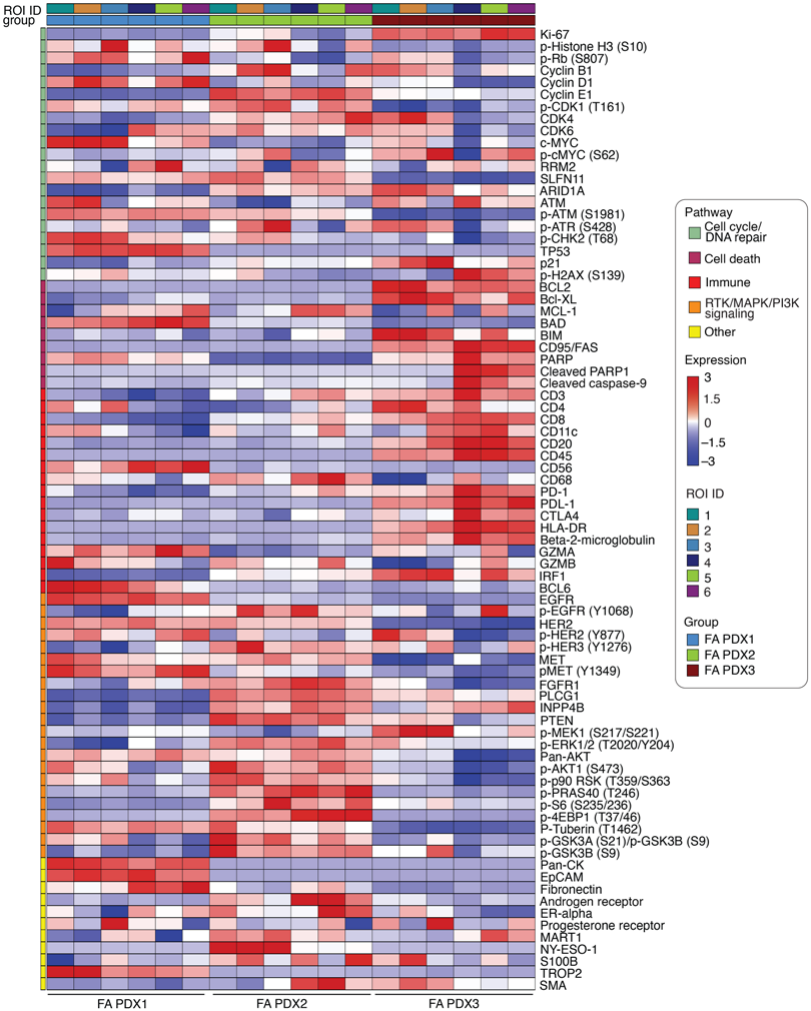
Supervised heatmap representation of DSP of protein targets across PDX samples. The heatmap illustrates the variation in expression levels of DSP protein targets across PDX samples, with 6 ROIs analyzed per PDX tumor. Columns correspond to individual samples, while the rows represent the protein targets for DSP. Each cell in the heatmap corresponds to the expression level of a specific protein target in a particular sample, with color intensity indicating the expression level (red for high expression, blue for low expression). Additionally, the heatmap is annotated with information regarding the pathways, groups, and ROI unique identifiers (ROI IDs) associated with each sample. Proteins are grouped according to their associated pathways.

Digital spatial profiling (DSP) (18, 19) was used to examine the expression of 81 proteins and phospho-proteins involved in cellular proliferation or survival. For each PDX model, we performed profiling on 6 different tumor regions and generated heatmaps of protein expression (Figure 3). FA PDX 1 was characterized by high expression of epithelial markers (EpCam, cytokeratins), as well as by elevated expression of the EGFR/HER family members EGFR and HER2. FA PDX 2 was characterized by activation of the PI3K/AKT signaling pathway (high levels of phosphorylated AKT [p-AKT] and p-PRAS40), consistent with the presence of a PI3K-activating mutation in this model, whereas FA PDX 3 exhibited high levels of Ki67, p-MEK, and the apoptosis regulatory proteins B-cell lymphoma 2 (Bcl-2), Bcl-X_L_, and Bim. Unsupervised clustering of the DSP data showed that the different regions of the FA PDX 1 tumor clustered together, as did different regions of the FA PDX 2 tumor, while the FA PDX 3 tumor displayed intratumoral heterogeneity, characterized by 2 distinct regions (Supplemental Figure 4). Collectively, these data demonstrated unique differences among the 3 FA-HNSCC PDX models, as well as substantial intratumoral heterogeneity in 1 model, confirming the necessity of personalized therapeutic approaches in this disease.

### FA-HNSCC PDX models mimic heightened cisplatin sensitivity characteristic of FA.

**Figure 4:**
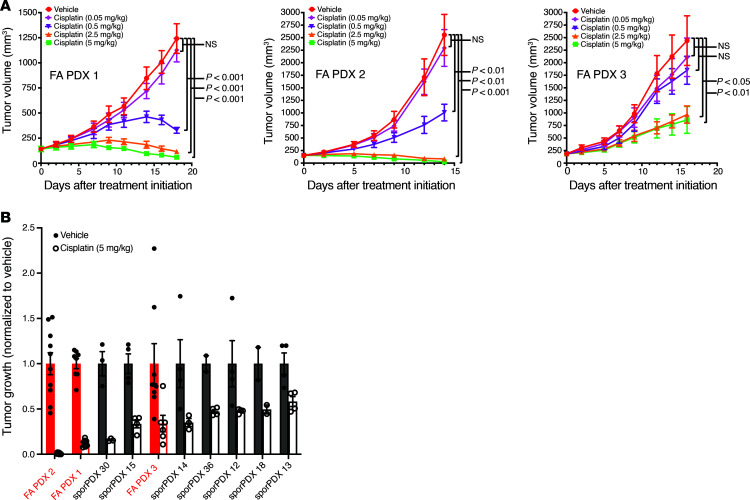
FA-HNSCC PDX exhibits heightened sensitivity to cisplatin. (**A**) Cisplatin dose response of FA-HNSCC PDX tumors. Mice harboring FA PDX 1, FA PDX 2, or FA PDX 3 tumors were treated once a week via i.p. injection with vehicle (saline) or varying doses of cisplatin (4 mice/group for FA PDX 1 and FA PDX 3; 5 mice/group for FA PDX 2; 2 tumors/mouse for all). Data represent the mean + SEM. Differences at the final time point in average tumor volumes between treatment groups were determined using Dunnett’s 2-tailed *t* test ad hoc to control the experiment-wise type I error rate at 5%. (**B**) Comparison of cisplatin sensitivity in FA-HNSCC PDX models (red) versus sporadic (spor) HNSCC PDX models. The average tumor volume on day 14 after cisplatin treatment (normalized to vehicle treatment) for the FA-HNSCC PDX is compared with the responses we have previously reported for 7 sporadic HNSCC PDX models (20). Mice harboring FA-HNSCC PDX tumors were treated once a week via i.p. injection with vehicle (saline) or cisplatin (5 mg/kg) (4 mice/group for FA PDX 1 and FA PDX 3; 5 mice/group for FA PDX 2; 2 tumors/mouse for all). Data represent the mean + SEM.

Platinum chemotherapy remains the mainstay of systemic chemotherapy regimens for sporadic HNSCC. However, the germline defects in DNA repair that are characteristic of FA result in extreme cellular sensitivity to this DNA-crosslinking agent and excessive toxicity in patients with FA (11). To test our prediction that the FA-HNSCC PDXs would exhibit heightened sensitivity to cisplatin, we assessed tumor growth following once-weekly treatment with cisplatin (Figure 4A). We observed potent, dose-dependent inhibition of FA PDX 1 and FA PDX 2 tumors, with tumor regression occurring at the higher doses of 2.5 and 5.0 mg/kg cisplatin. FA PDX 3 tumors, although responsive to cisplatin, were more resistant, and regression was not observed in this model. We have previously reported the response of 7 well-characterized, non-FA, sporadic HNSCC PDX models to a 14-day treatment with cisplatin (5 mg/kg) (20). Here, we treated mice harboring FA PDX 1, FA PDX 2, or FA PDX 3 tumors with cisplatin (5 mg/kg) for 14 days and compared the growth inhibition in our FA-HNSCC PDX models with the growth inhibition we previously reported in non-FA, sporadic HNSCC PDX models (Figure 4B). We found that the FA PDX 1 and FA PDX 2 tumors were the most sensitive to cisplatin in this comparison, while the FA PDX 3 tumor displayed moderate sensitivity (Figure 4B). These findings demonstrated that the phenotypic response of the FA PDX tumors to a DNA-damaging agent was consistent with what would be predicted for an FA-deficient tumor, with some heterogeneity in the response.

It is noteworthy that the FA PDX 1 model and FA PDX 2 and the corresponding primary patient tumors exhibited heterozygous loss of the gene encoding the DNA damage response (DDR) kinase ATM (Figure 2). It has been reported that inhibition of ATR, an alternative DDR kinase, can dramatically increase sensitivity to cisplatin in ATM-deficient tumors (21, 22), providing an opportunity for lowering the dose of cisplatin needed to achieve tumor growth inhibition. Although inhibition of ATR using AZD738 failed to enhance sensitivity to cisplatin in the FA PDX 1 model, we observed a modest enhancement of cisplatin sensitivity following ATR inhibition in the FA PDX 2 model (Supplemental Figure 5).

### Overexpression of activated EGFR in FA PDX 1 is associated with high sensitivity to cetuximab.

Cetuximab, a monoclonal antibody targeting EGFR, was approved by the FDA in 2006 for the treatment of sporadic HNSCC. However, neither expression nor activation (phosphorylation) of EGFR has consistently been shown to be a predictive biomarker of response, and EGFR tumor testing is not required for cetuximab administration. A small number of patients with FA HNSCC (*n* = 10) have been reported to have been safely treated with cetuximab, but there is no information on the EGFR status in the patients’ tumors (11, 14, 23–25).

**Figure 5:**
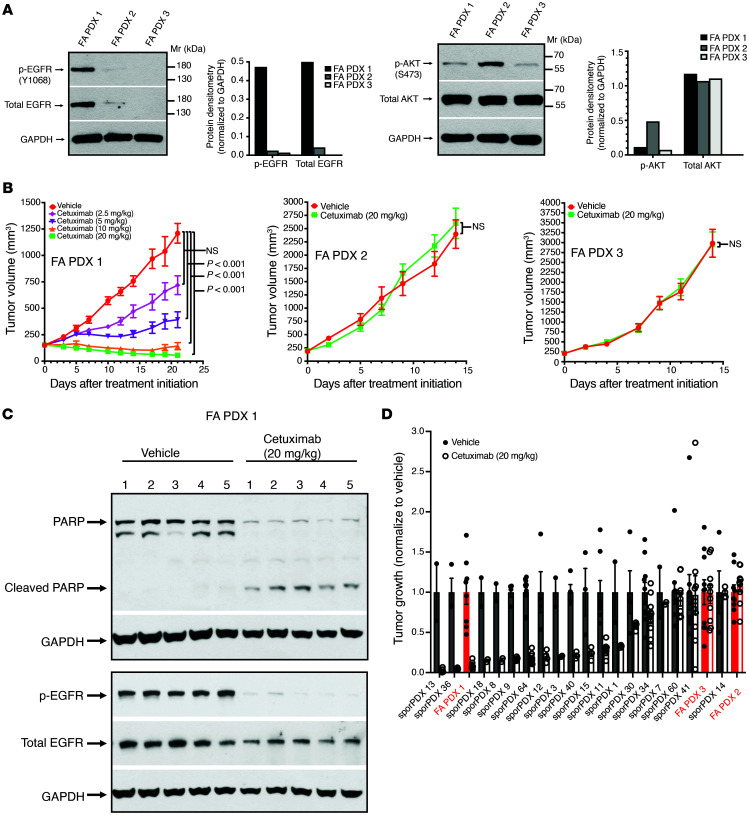
FA PDX 1 expresses high levels of EGFR and is highly sensitive to cetuximab. (**A**) Overexpression of total EGFR and p-EGFR by FA PDX 1, FA PDX 1, FA PDX 2, and FA PDX 3 tumors was analyzed. Tumor lysates were subjected to immunoblotting for p-EGFR (Y1068), total EGFR, p-AKT (S473), total AKT or GAPDH. (**B**) Dose-dependent inhibition of FA PDX 1, but not FA PDX 2 or FA PDX 3, by cetuximab. Mice with FA-HNSCC PDX tumors (5 mice/group, 2 tumors/mouse) were treated twice per week via i.p. injection with vehicle (saline) or varying doses of cetuximab (5 mice/group, 2 tumors/mouse). Tumor growth data for FA PDX 2 and FA PDX 3 are shown only for the highest cetuximab dose (20 mg/kg). Data represent the mean + SEM. Differences at the final time point in average tumor volumes between treatment groups were determined using Dunnett’s 2-tailed *t* test ad hoc to control the experiment-wise type I error rate at 5%. (**C**) PARP cleavage and loss of p-EGFR in cetuximab-treated FA PDX 1 tumors. Mice harboring FA PDX 1 tumors were treated twice weekly via i.p. injection with vehicle (saline) or cetuximab (20 mg/kg), and tumors were harvested on day 21. Tumor lysates were subjected to immunoblotting for PARP, p-EGFR, total EGFR, or GAPDH. The blot shows 1 tumor from each mouse. (**D**) Comparison of FA-HNSCC PDX models (red) versus sporadic HNSCC PDX models. The average tumor volume on day 14 after cetuximab treatment (normalized to vehicle) for the FA-HNSCC PDX models is compared with the responses we have previously reported for 18 sporadic HNSCC PDXs (26). Mice with FA-HNSCC PDX tumors were treated twice per week via i.p. injection with vehicle (saline) or cetuximab (20 mg/kg) (5 mice/group, 2 tumors/mouse). Data represent the mean + SEM. Mr, molecular weight.

Genomic analysis demonstrated amplification of *EGFR* in the FA PDX 1 model, but not in the other models, and DSP indicated elevated levels of EGFR protein in FA PDX 1. We therefore performed immunoblotting to rigorously assess the levels of total EGFR and p-EGFR (Y1068) in the FA-HNSCC PDX models. We found that both total EGFR and p-EGFR were expressed at high levels in FA PDX 1, consistent with the genomic and DSP findings for this model (Figure 5A). In contrast, the FA PDX 2 and FA PDX 3 models expressed only low or undetectable levels of total EGFR and p-EGFR. Immunoblotting of late-passage tumors (passages 16, 28, and 23 for FA PDX 1, FA PDX 2, and FA PDX 3, respectively) revealed that FA PDX 1 tumors retained high levels of total EGFR and p-EGFR expression relative to the other models (Supplemental Figure 6).

We next tested the predictive value of total EGFR and p-EGFR levels for the response to cetuximab in our FA-HNSCC PDX models and observed a striking correlation. Mice harboring FA-HNSCC PDX tumors were treated with cetuximab twice weekly via i.p. injection, and tumor growth was monitored (Figure 5B). Treatment of FA PDX 1 led to potent, dose-dependent inhibition of tumor growth, with regression observed at the highest doses of 10 or 20 mg/kg. Treatment of FA PDX 1 with cetuximab at the 20 mg/kg dose was associated with loss of full-length PARP protein and the appearance of cleaved PARP in the tumor specimens, indicative of apoptosis induction, as well as with loss of p-EGFR and a reduction in total EGFR levels (Figure 5C). In contrast to FA PDX 1, the FA PDX 2 and FA PDX 3 models, which lacked robust levels of total and p-EGFR, failed to respond to the highest cetuximab dose (20 mg/kg) (Figure 5B).

We have previously reported the effect of cetuximab treatment (20 mg/kg, twice weekly for 14 days) on the growth of 18 non-FA, sporadic HNSCC PDX models (26). Here, we treated the 3 FA-HNSCC PDX models similarly and compared the observed growth inhibition with that reported for the non-FA, sporadic HNSCC PDX models (Figure 5D). While FA PDX 2 and FA PDX 3 were among the least responsive to cetuximab in this comparison, FA PDX 1 was among the most sensitive. Collectively, our findings in the 3 FA-HNSCC PDX models showed that expression of total EGFR and/or p-EGFR predicted the response to the FDA-approved cetuximab, supporting the utility of further exploration of this agent in patients with FA-HNSCC whose tumors express high EGFR/p-EGFR levels.

**Figure 6:**
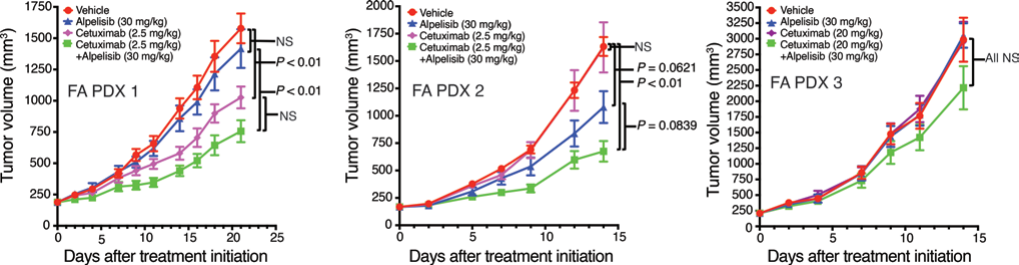
Combination treatment with cetuximab and the PI3K inhibitor alpelisib (BYL719). Mice with FA-HNSCC PDX tumors were treated with vehicle (i.p. saline for cetuximab; oral gavage of 0.5% methylcellulose + 0.1% Tween 80 for alpelisib); alpelisib (30 mg/kg, 5 times per week, oral gavage), cetuximab (2.5 mg/kg for FA PDX 1 and FA PDX 2, 20 mg/kg for FA PDX 3, i.p. twice/week); or the combination of alpelisib and cetuximab (5 mice/group, 2 tumors/mouse). Data represent the mean + SEM. Differences at the final time point in average tumor volumes between treatment groups were determined using Dunnett’s 2-tailed *t* test ad hoc to control the experiment-wise type I error rate at 5%.

The growth-promoting effects of EGFR activation are mediated, in large part, via activation of the PI3K/AKT signaling pathway. We and others have previously reported that the *PIK3CA* gene, encoding PI3K, is the most commonly altered oncogene in sporadic HNSCC (27, 28), with both gene amplification and mutation contributing to aberrant PI3K activity in tumor cells. Genomics analyses revealed *PIK3CA* amplification in FA PDX 1 (Figures 1 and 2). An increased *PIK3CA* copy number and a canonical activating mutation of *PIK3CA* (H1047R) were present in FA PDX 2, as well as in the corresponding primary tumor, and an increased *PIK3CA* copy number, albeit one that did not reach the amplification threshold, was present in FA PDX 3 (Figures 1 and 2, and Supplemental Tables 2 and 3). DSP data indicated hyperactivation of the PI3K/AKT pathway in FA PDX 2 (Figure 3), and immunoblotting confirmed elevated levels of phosphorylated/activated (S473) AKT (p-AKT) in this model in both early (Figure 5A) and late (Supplemental Figure 6) tumor passages, consistent with the presence of an activating *PIK3CA* mutation. Lower p-AKT levels were detected in the FA PDX 1 and FA PDX 3 models (Figure 5A and Supplemental Figure 6). We assessed the effect of inhibition of the PI3K/AKT pathway on tumor growth by treating the tumors with alpelisib (BYL719), a PI3K inhibitor that is FDA approved for use in *PIK3CA*-mutant breast cancer. Mice harboring the FA-HNSCC PDX tumors were treated with alpelisib (30 mg/kg via oral gavage, 5 times/week) alone or in combination with cetuximab (i.p., twice/week; 2.5 mg/kg for FA PDX 1 and FA PDX 2; 20 mg/kg for FA PDX 3) (Figure 6). As monotherapy, treatment with alpelisib reduced tumor growth only in the *PIK3CA*-mutant FA PDX 2 model. Similarly, treatment with the combination of alpelisib plus cetuximab demonstrated a trend toward additive tumor growth inhibition compared with either agent alone only in the FA PDX 2 model. Thus, although alpelisib remains investigational in sporadic HNSCC, in patients with FA-HNSCC whose tumors harbor an activating *PIK3CA* mutation, the addition of a PI3K inhibitor to a treatment regimen containing cetuximab may prove clinically beneficial.

### FA PDX 1 is highly sensitive to the pan-EGFR/HER family tyrosine kinase inhibitor dacomitinib.

**Figure 7:**
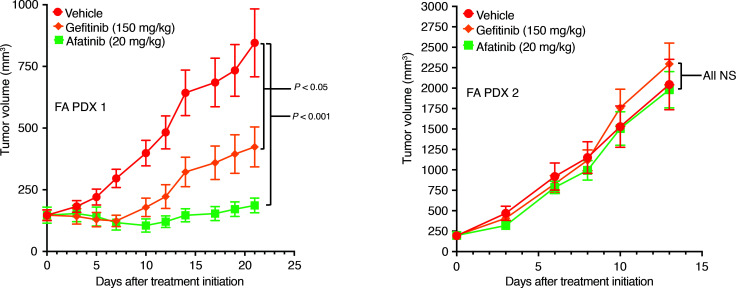
Gefitinib and afatinib inhibit the growth of FA PDX 1 tumors, but not FA PDX 2 tumors. Mice with FA-HNSCC PDX tumors were treated 5 times per week via oral gavage with vehicle (0.5% methylcellulose, 0.1% Tween 80); gefitinib (150 mg/kg); or afatinib (20 mg/kg) (4 mice/group for FA PDX 1; 5 mice/group for FA PDX 3; 2 tumors/mouse for all). Data represent the mean + SEM. Differences at the final time point in average tumor volumes between treatment groups were determined using Dunnett’s 2-tailed *t* test ad hoc to control the experiment-wise type I error rate at 5%.

The tyrosine kinase inhibitor (TKI) gefitinib targets EGFR, while the TKI afatinib targets both EGFR and the EGFR/HER family member HER2. Both agents have failed in unselected sporadic HNSCC cohorts and neither is FDA approved for this disease. Despite this, Montanuy et al. (29) have reported growth inhibition of xenograft tumors derived from FA-HNSCC cell lines deficient in the FA genes *FANCC* or *FANCL* following treatment with gefitinib or afatinib, prompting the development of a pending European trial of afatinib treatment of patients with FA-HNSCC (30). We therefore evaluated sensitivities to gefitinib and afatinib in 2 of the FA-HNSCC PDX models, both of which harbor altered *FANCA*, the most common FA gene alteration found in FA-HNSCC (16). Treatment of the FA PDX 1 model (high levels of total EGFR and p-EGFR) with gefitinib (150 mg/kg via oral gavage, 5 times/week) or afatinib (20 mg/kg, via oral gavage, 5 times/week) led to significant inhibition of tumor growth, with afatinib exhibiting greater potency (Figure 7). Treatment of the FA PDX 2 model (low levels of total EGFR and undetectable p-EGFR) with gefitinib or afatinib, however, failed to affect tumor growth.

**Figure 8:**
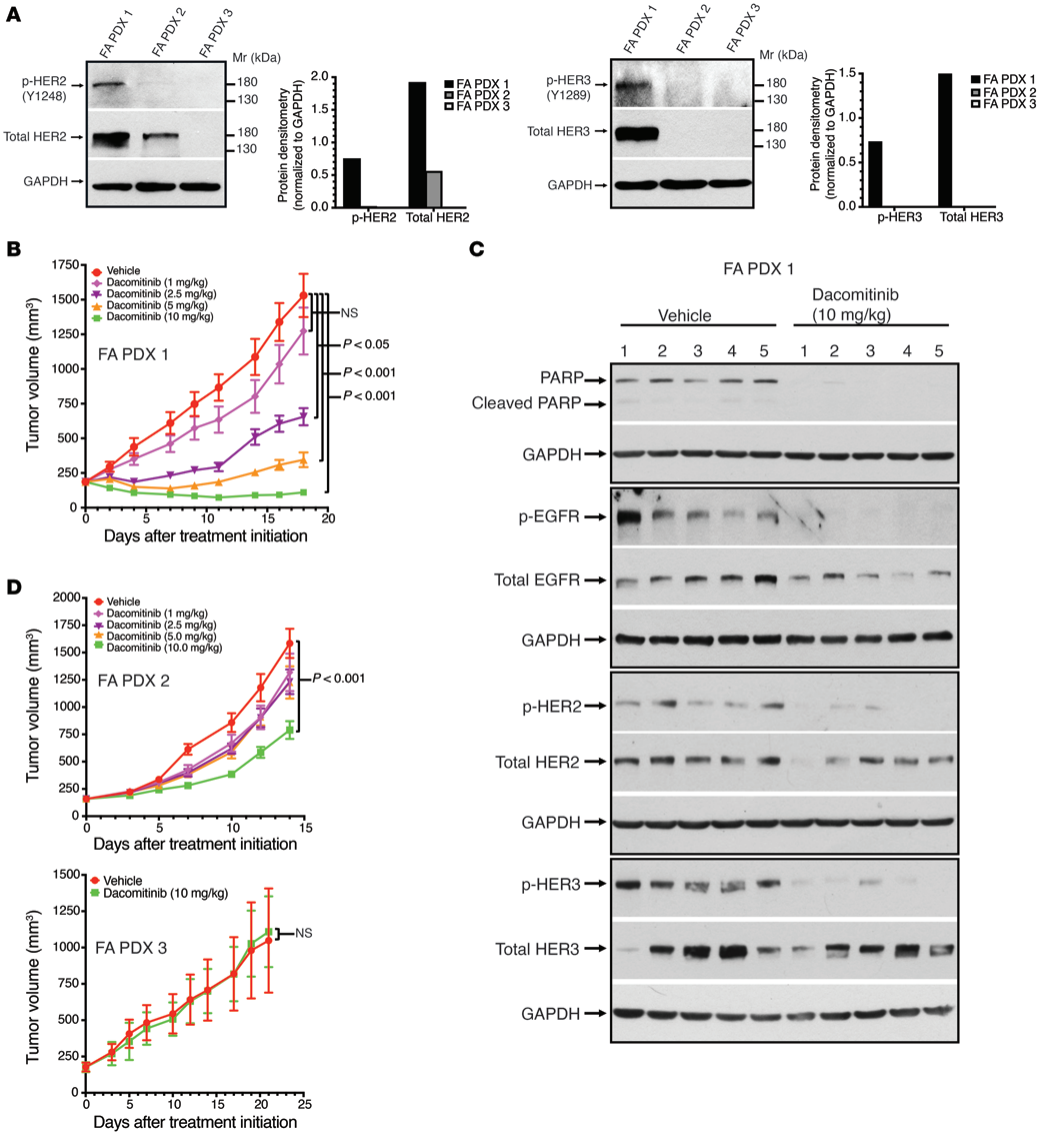
The pan-EGFR/HER TKI dacomitinib potently inhibits FA PDX 1 tumor growth. (**A**) Expression of total and phosphorylated forms of HER2 and HER3. FA PDX 1, FA PDX 2, and FA PDX 3 tumor lysates were subjected to immunoblotting for p-HER2 (Y1248), total HER2, p-HER3 (Y1289), total HER3, or GAPDH. (**B**) Dose-dependent inhibition of FA PDX 1 tumors by dacomitinib. Mice with FA PDX 1 tumors were treated with vehicle (0.5% methycellulose + 0.1% Tween 80) or varying doses of dacomitinib via oral gavage 5 times per week (5 mice/group, 2 tumors/mouse). Data represent the mean + SEM. Differences at the final time point in average tumor volumes between treatment groups were determined using Dunnett’s 2-tailed *t* test ad hoc to control the experiment-wise type I error rate at 5%. (**C**) Loss of full-length PARP and phosphorylated forms of EGFR, HER2, and HER3 in dacomitinib-treated FA PDX 1 tumors. Tumors from the treated mice in **B** were harvested on day 18, and tumor lysates were subjected to immunoblotting for PARP, p-EGFR, total EGFR, p-HER2, total HER2, p-HER3, total HER3 and GAPDH. The blot shows 1 tumor from each mouse. (**D**) Dacomitinib insensitivity of FA PDX 2 and FA PDX 3 tumors. Mice harboring FA PDX 2 or FA PDX 3 tumors were treated 5 times per week via oral gavage with vehicle or dacomitinib (10 mg/kg). Data represent the mean + SEM. Statistical differences were determined as in **B**.

The high expression levels of total EGFR and p-EGFR in FA PDX 1 and the associated sensitivity of this model to EGFR inhibitors (gefitinib, afatinib, and cetuximab) prompted us to examine the expression of other members of the EGFR/HER protein family in all 3 FA-HNSCC PDX models. As shown in Figure 8A, total HER2 was expressed at high levels in FA PDX 1, at lower levels in FA PDX 2, and was undetectable in FA PDX 3 models. FA PDX 1 also expressed p-HER2 (Y1248). Notably, total HER3 and p-HER3 (Y1289) were highly expressed by FA PDX 1, but not by the other models. In view of the expression, albeit variable, of additional EGFR/HER family members, we sought to determine the effect of pan-EGFR/HER family inhibition. Dacomitinib is an irreversible pan-EGFR/HER TKI that is approved by the FDA for use in lung cancer and is under active clinical investigation in sporadic HNSCC (NCT01449201, NCT00768664) (31–33). Treatment with dacomitinib (5 times/week via oral gavage) resulted in potent dose-dependent inhibition of tumor growth in the FA PDX 1 model (Figure 8B). At the highest dose (10 mg/kg), we observed tumor regression, accompanied by loss of full-length PARP, p-EGFR, p-HER2, and p-HER3 (Figure 8C). By contrast, FA PDX 3 tumors failed to respond to treatment with 10 mg/kg dacomitinib (Figure 8D). We observed a moderate, though statistically significant, response to dacomitinib in the FA PDX 2 model, consistent with increased expression of HER2 in this model (Figure 8, A and D).

### Targeting antiapoptotic proteins inhibits the growth of Bcl-2–overexpressing FA PDX 3.

**Figure 9:**
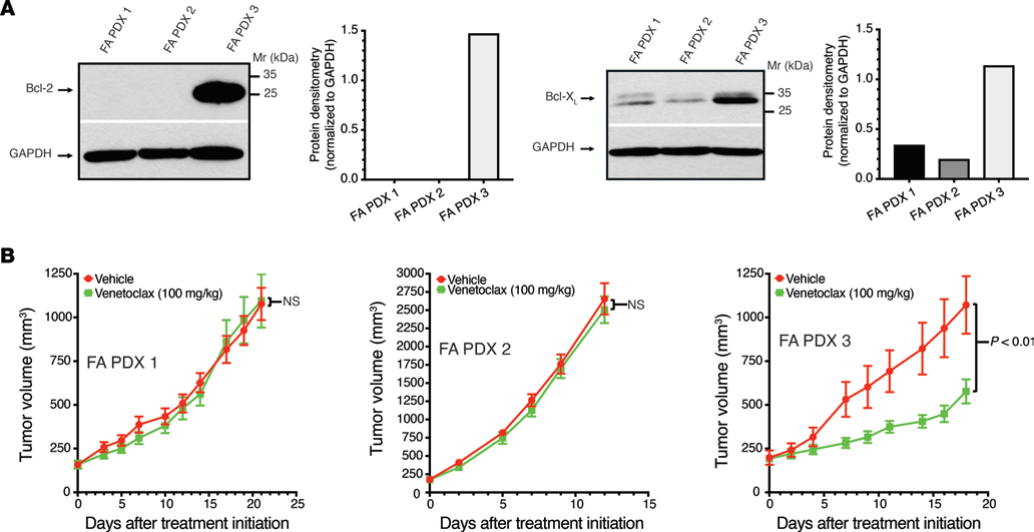
The Bcl-2 inhibitor venetoclax inhibits FA PDX 3 tumor growth. (**A**) Expression of Bcl-2 and Bcl-X_L_ by FA PDX 3. FA-HNSCC PDX tumor lysates were subjected to immunoblotting for Bcl-2, Bcl-X_L_, or GAPDH. (**B**) Inhibition of FA PDX 3 tumor growth by venetoclax. Mice with FA-HNSCC PDX tumors were treated 5 times per week with vehicle (0.5% methycellulose + 0.1% Tween 80) or venetoclax (100 mg/kg) via oral gavage (5 mice/group, 2 tumors/mouse). Data represent the mean + SEM. Differences at the final time point in average tumor volumes between treatment groups were determined using Dunnett’s 2-tailed *t* test ad hoc to control the experiment-wise type I error rate at 5%.

The FA PDX 3 tumor model exhibited greater resistance to cisplatin relative to the other FA-HNSCC PDX models and did not respond to EGFR/HER-targeting agents or the PI3K inhibitor alpelisib. Tumor resistance to DNA-damaging agents, as well as molecular targeting agents, can be due to overexpression of the antiapoptotic Bcl-2 family members Bcl-2 and Bcl-X_L_, which were noted to be expressed at high levels in FA PDX 3 tumors in our DSP studies (Figure 3). Immunoblotting confirmed markedly elevated expression of Bcl-2 and modestly elevated Bcl-X_L_ expression in FA PDX 3 tumors compared with the other 2 models in both early (Figure 9A) and late (Supplemental Figure 6) tumor passages. We therefore examined the effect of venetoclax, an FDA-approved Bcl-2 inhibitor, on FA PDX 3 tumor growth. Treatment with venetoclax (100 mg/kg, 5 times/week via oral gavage) led to growth inhibition in this drug-resistant FA-HNSCC tumor model (Figure 9B and Supplemental Figure 7). The antitumor effects of venetoclax in this FA-HNSCC PDX model identifies a unique personalized therapeutic approach for FA-associated HNSCC that is based on the molecular characterization of our preclinical models.

## Discussion

FA is a rare genetic condition (1–9 cases per million individuals worldwide). Improvements in managing bone marrow failure in childhood has led to much-improved survival to adulthood of individuals with FA. These patients are now developing solid tumors, mostly HNSCCs, at an alarming rate (700-fold increased risk). If surgical resection is not curative, these cancers are lethal, given the intolerance of most cytotoxic chemotherapy agents and radiation toxicity in these patients, even when administered judiciously. The present work describes what we believe to be the only FA PDX models in the world, and our results reveal new targets (e.g., Bcl-2) and predictive biomarkers for FDA-approved agents (e.g., cetuximab). The testing of potential therapeutic agents in these PDX models may lead to improved outcomes for patients with FA-HNSCC.

Clinical trials in rare diseases like FA-HNSCC are challenging because of the relatively small number of patients, hindering accrual and limiting the number of different agents that can be tested. In addition, the germline repair defects that characterize FA preclude the use of cisplatin, which is the most effective chemotherapy for sporadic HNSCC. While radiation can be safely administered in most cases, dose reductions and treatment breaks are often required (11). Immune checkpoint inhibitors are considered by many to be contraindicated in the setting of prior bone marrow transplantation. Since most patients with FA undergo hematopoietic stem cell transplantation to manage their bone marrow disease as children, clinicians are reluctant to administer the immune checkpoint inhibitors pembrolizumab or nivolumab in FA-HNSCC, although they are approved for use in sporadic HNSCC.

The development of preclinical models that closely resemble patient tumors can both accelerate the identification of new targets and facilitate the clinical development of novel agents. For many cancers, including HNSCC, implanting the patient’s tumor directly into a mouse allows for the generation of an in vivo model that can be propagated for testing candidate agents including drug combinations. This enables identification of promising therapeutic agents and strategies while also facilitating prioritization among different drugs.

It is the rare oncologist who sees even a single patient with FA-HNSCC over the course of their career. When a clinician encounters an individual with FA-HNSCC, they are understandably reluctant to administer standard chemoradiation, which is associated with substantial dose-limiting toxicities in patients with FA. To test and prioritize candidate targeted agents, we developed 3 robust PDX models from patients with FA-HNSCC. Genomic characterization of the models confirmed their close resemblance to the patients’ tumors from which they were derived. Two of the 3 models were highly sensitive to cisplatin compared with PDXs from patients with sporadic HNSCC. Successful delivery of low-dose cisplatin has been previously reported in a patient with FA with esophageal squamous cell carcinoma (34). However, administration of cisplatin to a patient with FA-HNSCC would have to be carefully considered, given the known toxicities in this population.

Sporadic HNSCC is widely characterized by EGFR overexpression. Given the target abundance and antitumor activity in preclinical xenograft models, EGFR inhibitors, most commonly monoclonal antibodies and TKIs, have been widely tested in patients with sporadic HNSCC. To date, the IgG1 monoclonal antibody cetuximab is the only EGFR inhibitor to receive FDA approval, although there is no evidence that the effectiveness of cetuximab correlates with EGFR expression levels in the tumor. EGFR TKIs have failed to improve survival compared with standard of care in phase III trials conducted in unselected patients with HNSCC, despite showing activity in the setting of some candidate predictive biomarkers (35).

Cetuximab has been safely administered to patients with FA-HNSCC, so we compared the activity of cetuximab in our FA-HNSCC PDXs with its effect in PDXs from patients with sporadic HNSCC. Only 1 of the FA PDX models (FA PDX 1) expressed high levels of EGFR/p-EGFR, and it was exquisitely sensitive to cetuximab. In contrast, EGFR was expressed at only negligible levels in the other 2 FA-HNSCC PDXs, both of which were resistant to this drug in vivo. Given the relative ease of measuring EGFR expression in most clinical laboratories, it would be appealing to select patients with FA-HNSCC for cetuximab therapy on the basis of expression of the target in their tumor.

FA PDX 1 also expressed other members of the EGFR family, so we tested the effect of dacomitinib, a pan-EGFR/HER TKI that is FDA approved for non–small cell lung cancer and is in clinical development in HNSCC. We found that FA PDX 1 was highly sensitive to dacomitinib, with evidence of tumor regression. Dacomitinib was less effective (FA PDX 2) or completely inactive (FA PDX 3) against the other tumors models, which expressed lower EGFR levels compared with FA PDX 1.

*PIK3CA* is the most commonly mutated oncogene in sporadic HNSCC, and alpelisib, a small-molecule targeting this oncogene, has been approved by the FDA for patients with breast cancer who have specific genetic alterations in their tumor (28, 36). Genomic analyses revealed *PIK3CA* amplification in FA PDX 1 and an increased *PIK3CA* copy number as well as a canonical activating mutation of *PIK3CA* (H1047R) in FA PDX 2. Alpelisib was most active in FA PDX 2, showing both single-agent activity as well as a trend toward enhancement of cetuximab’s effects. In contrast, there was no activity of alpelisib in FA PDX 3, which harbored a nonstatistically significant increase in the *PIK3CA* copy number. While, to our knowledge, there are no reports of patients with FA-HNSCC receiving alpelisib therapy, there is no obvious reason that drug toxicity should be increased in patients with FA. These findings are immediately translatable, given that alpelisib is FDA approved for breast cancers with selected *PIK3CA* mutations.

One of the FA PDX models was stubbornly resistant to nearly all agents tested (FA PDX 3). Protein profiling indicated that this PDX uniquely expressed high levels of the antiapoptotic protein Bcl-2. Bcl-2 inhibitors such as venetoclax are FDA approved for treating several types of leukemias, with no evidence of activity in most solid tumors, including HNSCC (37). Venetoclax showed antitumor efficacy in this FA-HNSCC PDX model only. Since the patient whose tumor generated this model is still in remission, these findings immediately suggest that a Bcl-2 inhibitor could be justified if this patient develops disseminated disease.

N-of-1 clinical trial designs are increasingly conducted in the setting of rare diseases including cancers. HNSCC arising in an individual with FA represents one of the rarest of conditions, with no clinical trial platform and legitimate concerns about the tolerability of regional or systemic therapies that have been approved for sporadic HNSCC. Here, we report the first, to our knowledge, preclinical in vivo models from FA-HNSCC tumors. These models can be characterized and deployed to test and prioritize precision medicine strategies. Limitations include the relatively small number of FA-HNSCC PDX models, the lack of an intact immune system in the PDX-bearing mice, and the potential toxicity of any agent in a patient with FA. Our findings confirm the biologic heterogeneity of FA-HNSCC and suggest that assessment of some standard biomarkers such as EGFR, EGFR family proteins, PI3K activation, and Bcl-2 expression should be considered to inform the management of patients with this rare and lethal disease.

## Methods

### Sex as a biological variable.

Our study examined human PDX tumors exclusively in female mice. It is unknown whether the findings are relevant for male mice.

### Generation of FA-HNSCC PDX models.

For FA PDX 1, following surgical resection of the patient’s tumor, the tumor specimen was disinfected first with 100% ethanol, then with antibiotic-antimycotic (Gibco, Thermo Fisher Scientific) containing 1% ceftazidime (Hospira Worldwide/Pfizer), and finally with PBS (Gibco, Thermo Fisher Scientific) containing 1% ceftazidime. After disinfection, a small piece of the tumor was viably frozen in freezing media (20% DMSO, 60% FCS, 20% DMEM) and delivered on dry ice to the laboratory at UCSF. The frozen tumor specimen was subsequently thawed and washed twice with ice-cold RPMI-1640 media (Gibco, Thermo Fisher Scientific) prior to implantation. For implantation into mice, the tumor specimen was dissected into pieces approximately of 2 mm^3^ in size, coated with Matrigel (Corning), and implanted s.c. into both flanks of 5- to 6-week-old female NOD.Cg-*Prkdc^scid^ Il2rg^tm1Wjl^*/SzJ (NSG) mice (The Jackson Laboratory).

For FA PDX 2 and FA PDX 3, resected tumor tissue was placed in HypoThermosol solution (BioLife Solutions) and transported at 4°C to the laboratory. Following previously described procedures (38), the tissue was minced, mixed (50:50) with Matrigel, and implanted s.c. into 6- to 8-week-old female NSG mice (The Jackson Laboratory) to generate the PDX models. Mice were monitored daily, and models were transplanted into mice 3 times before being deemed established.

For histological analyses, PDX tumor tissues were harvested from euthanized mice, placed into cassettes, and submersed in 10% neutral buffered formalin (MilliporeSigma) for 24 hours. The cassettes with fixed tissues were then transferred into 70% ethanol. The tissues were subsequently embedded in paraffin, sectioned into 5 μm sections, and subjected to H&E staining by the Histology and Light Microscopy Core of the UCSF Gladstone Institute. Images were taken using a Keyence microscope.

### Passage and maintenance of FA-HNSCC PDX models.

The established FA-HNSCC PDX tumors were maintained in NSG mice by routine passaging. Briefly, when tumors reached 2 cm in diameter, the mice were euthanized and the tumors harvested. The tumor specimens were immediately placed in antibiotic-antimycotic for 5 minutes and then washed with RPMI 1640 medium (Gibco, Thermo Fisher Scientific). The tumors were then cut into 2 mm^3^ pieces and implanted s.c. into both sides of 5- to 6-week-old female NSG mice. For cryopreservation of tumor specimens, freshly harvested tumors were cut into pieces of approximately 2 mm^3^, and the pieces were viably frozen in RPMI 1640 containing 10% FBS and 5% DMSO.

### DSP.

NanoString GeoMx DSP studies (18, 19) were performed to determine the in situ expression profile of up to 81 proteins on the 3 FA-HNSCC PDX tumor samples. All GeoMx DSP reagents and antibody panels were validated by NanoString Technologies, including ThermoFisher Syto13 dye, GeoMx DSP Collection Plates, the GeoMx Instrument Buffer Kit, the GeoMx FFPE slide preparation Kit, Master Kit-12 reactions, the GeoMx Hyb Code Pack, and protein panel R codes (NanoString Technologies). Additionally, a number of proteins from the Oregon Health and Science University (OHSU) custom panel were validated by the Knight Diagnostic Laboratories (OHSU, Portland, Oregon, USA).

Formalin-fixed, paraffin-embedded sections (5 μm) of PDX tumors were placed on Fisherbrand Superfrost Plus Microscope Slides and baked in a dry oven (Precision Scientific) overnight at 60°C. Slides were then deparaffinized, and antigen retrieval was performed according to the GeoMx–nCounter Slide Preparation User Manual (MAN-10087-08 p.18–p.24). Next, slides were incubated with antibody panels including panels for detection of human receptor tyrosine kinases, cell death signaling proteins, and PI3K/AKT pathway components, as well as an OHSU custom panel that was designed to detect cell-cycle and DNA damage pathway analytes. All samples were reviewed by a board-certified pathologist, and 6 regions of interest (ROIs) per sample were selected to encompass distinct tumor regions on each sample. Circle or geometric ROIs were drawn with a maximum ROI diameter of 660 μm^2^ or 660 × 785 μm^2^ for the collection. Morphological segmentation was not considered for this study due to the high tumor content of the PDX specimens. DNA-barcoded oligonucleotides from antibodies were cleaved by UV and collected in a 96-well plate. DNA samples were hybridized with color-barcoded code sets and protein panel R probe sets, which generated TagSets that were counted by the nCounter FLEX/MAX system (NanoString). The nCounter system generated a reporter code count (RCC) data file. RCC data were imported to DSP and normalized using the geometric mean. Samples were further analyzed using Rv4.2.2, and figures were generated using R package ggplot (version 3.4.2) and ComplexHeatmap packages, where the distance for heatmaps was calculated using Euclidean distance, and clustering was performed using complete linkage clustering.

### Treatment of FA-HNSCC PDX models.

For in vivo therapeutic studies, PDX tumors were implanted into both flanks of 5- to 6-week-old female NSG mice. When the tumor volumes reached approximately 150 mm^3^ in size, the mice were randomized into groups and treated with either vehicle or drug. Tumors were measured with calipers 3 times per week, and tumor volumes were calculated using the formula: volume = (length × width × width)/2.

For cisplatin (TCI America) treatments, mice were treated once per week via i.p. injection with vehicle (normal saline) or cisplatin (0.05, 0.5, 2.5, or 5 mg/kg). Experiments with FA PDX 1 and FA PDX 3 used 4 mice per group (2 tumors/mouse). Experiments with FA PDX 2 used 5 mice per group (2 tumors/mouse).

Studies with the ataxia telangiectasia and Rad3-related (ATR) protein inhibitor AZD6738 (Selleck Chemicals) involved treatment 5 days per week via oral gavage with vehicle (0.5% methylcellulose and 0.1% Tween 80 [MilliporeSigma]) or AZD6738 (5, 25, or 50 mg/kg). Studies with FA PDX 1 and FA PDX 2 used 5 mice per group with 2 tumors per mouse. For treatment with the combination of cisplatin plus AZD6738, mice were treated with vehicle (normal saline once per week by i.p. injection and 0.5% methylcellulose/0.1% Tween 80, 5 days/week via oral gavage); cisplatin alone (0.1 mg/kg once per week by i.p. injection); AZD6738 alone (25 mg/kg, 5 days/week by oral gavage); or the combination of cisplatin (0.1 mg/kg) and AZD6738 (25 mg/kg). Experiments with FA PDX 1 used 6 mice per group (2 tumors/mouse), and experiments with FA PDX 2 used 5 mice per group (2 tumors/mouse).

For studies with cetuximab (Eli Lilly), FA PDX 1 mice (5 mice/group, 2 tumors/mouse) were treated twice a week by i.p. injection with vehicle (normal saline) or cetuximab (2.5, 5, 10, or 20 mg/kg). FA PDX 2 and FA PDX 3 mice (5 mice/group, 2 tumors/mouse) were treated with vehicle (normal saline) or 20 mg/kg cetuximab. For treatment with the combination of the PI3K inhibitor alpelisib (BYL719; Selleck Chemicals) plus cetuximab, FA PDX1 and FA PDX 2, mice were treated with vehicle (normal saline 2 times/week by i.p. injection + 0.5% methylcellulose/0.1% Tween 80, 5 days/week by oral gavage); cetuximab alone (2.5 mg/kg, 2 times/week by i.p. injection); alpelisib alone (30 mg/kg, 5 days/week by oral gavage); or the combination of cetuximab (2.5 mg/kg) and alpelisib (30 mg/kg). FA PDX 3 mice (5 mice/group, 2 tumors/mouse) were treated with vehicle (normal saline 2 times/week by i.p. injection + 0.5% methylcellulose/0.1% Tween 80, 5 days/week via oral gavage); cetuximab alone (20 mg/kg, 2 times/week by i.p. injection); alpelisib alone (30 mg/kg, 5 days/week via oral gavage); or the combination of cetuximab (20 mg/kg) and alpelisib (30 mg/kg).

For treatments involving dacomitinib (MilliporeSigma), FA PDX 1 and FA PDX 2 mice (5 mice/group, 2 tumors/mouse) were treated 5 days per week via oral gavage with vehicle (0.5% methylcellulose/0.1% Tween 80) or dacomitinib (1, 2.5, 5, or 10 mg/kg). FA PDX 3, mice (5 mice/group, 2 tumors/mouse) were treated 5 days per week via oral gavage with vehicle (0.5% methylcellulose/0.1% Tween 80) or dacomitinib (10 mg/kg). For treatments involving gefitinb or afatinib (both from Selleck Chemical), mice were treated 5 days per week via oral gavage with vehicle (0.5% methylcellulose/0.1% Tween 80), gefitinib (150 mg/kg), or afatinib (20 mg/kg). Experiments with FA PDX 1 mice used 4 mice per group (2 tumors/mouse), and experiments with FA PDX 2 mice involved 5 mice per group (2 tumors/mouse). For venetoclax (MedChemExpress) treatments, tumor-bearing mice (5 mice/group, 2 tumors/mouse) were treated 5 days per week via oral gavage with vehicle (0.5% methylcellulose/0.1% Tween 80) or venetoclax (100 mg/kg).

### Immunoblotting.

Tumor tissues were homogenized in cell lysis buffer from Cell Signaling Technology (CST) containing phosSTOP Phosphatase Inhibitor Cocktail (Roche Diagnostics) and cOmplete Protease Inhibitor Cocktail (Roche Diagnostics). Proteins (30 μg/lane) were electrophoresed on 10% SDS/polyacrylamide gels and transferred to polyvinylidene difluoride membranes. The membranes were blocked for 1 hour at room temperature in TBST (10 mM Tris [pH 8.0], 150 mM NaCl, 0.1% Tween 20) with 5% nonfat milk, washed 3 times in TBST, and then incubated at 4°C overnight with primary antibodies in TBST containing 3% nonfat milk. Following 3 washes in TBST, the membranes were incubated for 1 hour at room temperature with secondary antibodies in TBST containing 3% nonfat milk. The membranes were then washed 3 times in TBST, and luminol reagent (Santa Cruz Biotechnology) was used to detect the immunoreactive bands. ImageJ (NIH) was used for densitometric analysis. The primary antibodies used in this study were as follows: anti–p-EGFR (CST, catalog 3777S); anti-EGFR (BD Transduction Laboratories, catalog 610017); anti–p-HER2 (CST, catalog 2244S); anti-HER2 (CST, catalog 4290S); anti–p-HER3 (CST, catalog 4561S); anti-HER3 (CST, catalog 12708S); anti–p-AKT (CST, catalog 4060S); anti-AKT (CST, catalog 4691S); anti-PARP (CST, catalog 9542S); anti–Bcl-2 (CST, catalog 3498T); anti–Bcl-X_L_ (CST, catalog 2764S); and anti-GAPDH (CST, catalog 5174S). The secondary antibodies used were: goat anti–mouse HRP (Bio-Rad, catalog 1706516) or goat anti–rabbit HRP (Bio-Rad, catalog 1706515).

### Genomics analysis.

DNA was extracted from samples using the Qiagen Blood and Cell Culture Extraction kit. Illumina whole-genome sequencing was performed at the NIH Intramural Sequencing Center using PCR-free TruSeq library prep. Tumor samples were sequenced to 60× genome coverage, and normal samples were sequenced to 30× genome coverage. For PDX data analysis, human and contaminating mouse DNA sequences were distinguished using BBSplit from the BBMap toolset (https://sourceforge.net/projects/bbmap/). In brief, reads were aligned with BBSplit against a combined reference database containing GRCh37 (human) and GRCm38 (mouse). Ambiguous sequence alignments were removed in case they could align equally well to the human and mouse reference genome.

Sequences determined to best fit the human genome using BBSplit were aligned against GRCh37 using BWA-mem2 (39) (using default settings). Variant calling, annotation, and filtering were performed as described in detail previously for tumors from patients with FA (16). Copy number variants (CNVs) for cases 1 and 2 were called from the allele-specific copy number analysis of tumors (ASCAT) output, and for case 3, the CNVkit (https://github.com/etal/cnvkit; v0.9.11; commit ID 450726e) was used for CNV calling (40). For case 3, an amplification was defined as a log_2_ fold change of greater than 0.6, and a deletion was defined as a log_2_ fold change of less than –1.

Initial genes of interest for mutational analysis for oncoplot generation were selected from The Cancer Genome Atlas (TCGA) as mutated in HNSCC (27). Additionally, genes previously identified as mutated in FA-HNSCC were added (16). Called variants, including structural variants (SVs), indels, CNVs, and SNVs, were filtered against the generated gene list, identified, and represented in oncoplots. Multiple colors were used if multiple types of variants were identified.

### Statistics.

Longitudinally measured tumor growth data were analyzed on the log-transformed scale using a random effects model whenever the model’s assumptions were satisfied. The random effects analysis compared treatments on the basis of their effects on the average rates of tumor growth within treatment groups while accounting for differential growth rates among individual mice. The significance of differences in average growth rates was tested using contrasts, and the Bonferroni method was applied to control the experiment-wide type I error rate, ensuring it did not exceed 5%. When the assumptions of the random-effects model were not met, a 1-way ANOVA was used to analyze the last measured tumor volume data on the log-transformed scale. Treatment groups were compared for differences in average tumor volume using the Dunnett’s 2-tailed *t* test ad hoc to control the experiment-wise type I error rate at 5%. Data represent the mean + SEM. Precise *P* values are given in the figures, with *P* < 0.05 considered significant.

### Study approval.

Tumor tissue was collected under an IRB-approved protocol (The Rockefeller University, protocol AAU-0112) after obtaining proper consent. Studies with mice were approved by the IACUC of UCSF (IACUC protocol AN202832), the IACUC of The Rockefeller University (IACUC protocol 20069-H), and the IACUC of MSKCC and the Research Animal Resource Center (IACUC protocol 04-03-009).

### Data availability.

PDX models are available pending completion of a material transfer agreement with The Rockefeller University. Human data, including Illumina whole-genome data from samples (3 PDXs, 3 matched normal samples, and 2 primary tumors) will be shared upon request with proper IRB approval and a data-sharing agreement. Complete processed data files are included as Supplemental Table 3 (https://github.com/MathijsSanders/SangerLCMFiltering, commit ID b482a0d, and https://github.com/MathijsSanders/AnnotateBRASS; commit ID 3ae7458). Values for all data points in the graphs are reported in the Supporting Data Values file.

## Author contributions

JRG, GBM, AS, and DEJ conceived experiments and reviewed and interpreted results. AS and MJ consented individuals and obtained patients’ primary tumor specimens. DIK, MU, TS, and MBW provided patients’ primary tumor specimens. AS and YCL obtained and reviewed clinical data. HL, YZ, IK, and BAH implanted tumor specimens and generated and maintained PDX models. EDS oversaw PDX generation at MSKCC. SRL performed and interpreted histology. SCC and FXD sequenced PDXs, primary tumors, and germline samples and analyzed germline variants. MAS, ALHW, BAH, and AS performed genomics analyses. JP, AN, JL, and GBM conducted and analyzed DSP. HL, YZ, and JGA performed in vivo therapeutic studies and immunoblotting. MOK performed statistical analyses. JRG, HL, RHL, AS, and DEJ wrote the original manuscript. All authors reviewed and had the opportunity to edit the manuscript.

## Funding support

This work is the result of NIH funding, in whole or in part, and is subject to the NIH Public Access Policy. Through acceptance of this federal funding, the NIH has been given a right to make the work publicly available in PubMed Central.

- Fanconi Cancer Foundation grants (to JRG, DEJ, and AS).
- NIH grants R35 CA231998 (to JRG), P30 CA008748 (to EDS), K99 HL150628 (to MJ), and UL1 TR001866 (to AS).
- Medical Scientist Training Program grant from a National Institute of General Medical Sciences (NIGMS), NIH award to the Weill Cornell/Rockefeller/Sloan Kettering Tri-Institutional MD-PhD Program (T32GM152349) (to BAH).
- Intramural Research Program of the NIH National Human Genome Research Institute (to SCC).
- V Foundation translational grant T2019-013 (to AS).
- Stand Up To Cancer–Fanconi Cancer Foundation–Farrah Fawcett Foundation Head and Neck Cancer Research Team Grant (to AS).
- KWF Kankerbestrijding Young Investigator Grant (12797/2019-2, Bas Mulder Award from the Dutch Cancer Foundation (to MAS).

## Supplementary Material

Supplemental data

Supplemental table 1

Supplemental table 2

Supplemental table 3

Supporting data values

## Figures and Tables

**Table 1:**
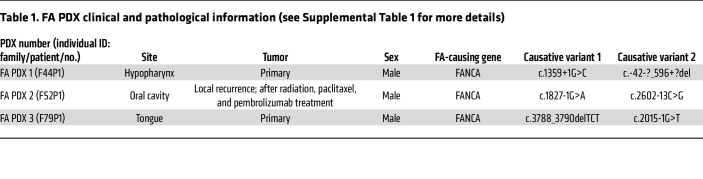
FA PDX clinical and pathological information (see Supplemental Table 1 for more details)

## Version 1 12/16/2025

In-Press Preview

## Version 2 02/16/2026

Electronic publication
