# Supplementary material for: Patient-derived xenograft models of Fanconi anemia–associated head and neck cancer identify personalized therapeutic strategies
Source: J Clin Invest. 2025 Dec 16;136(4):e195334. doi: 10.1172/JCI195334 (PMC12904715; doi:10.1172/JCI195334)
Supplement: Supplemental data [file jci-136-195334-s131.pdf]

Supplemental Figure 1

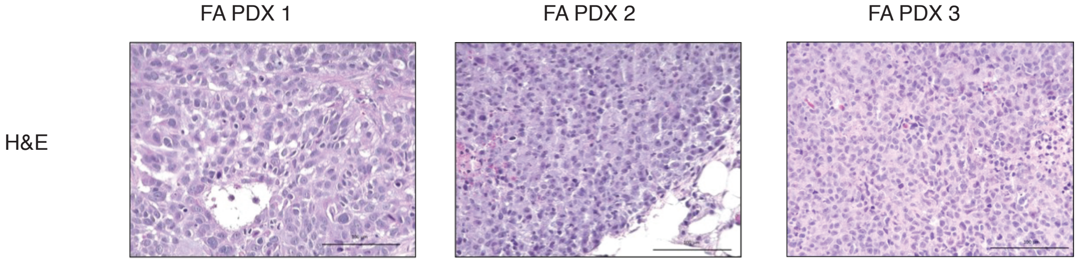

Supplemental Figure 2

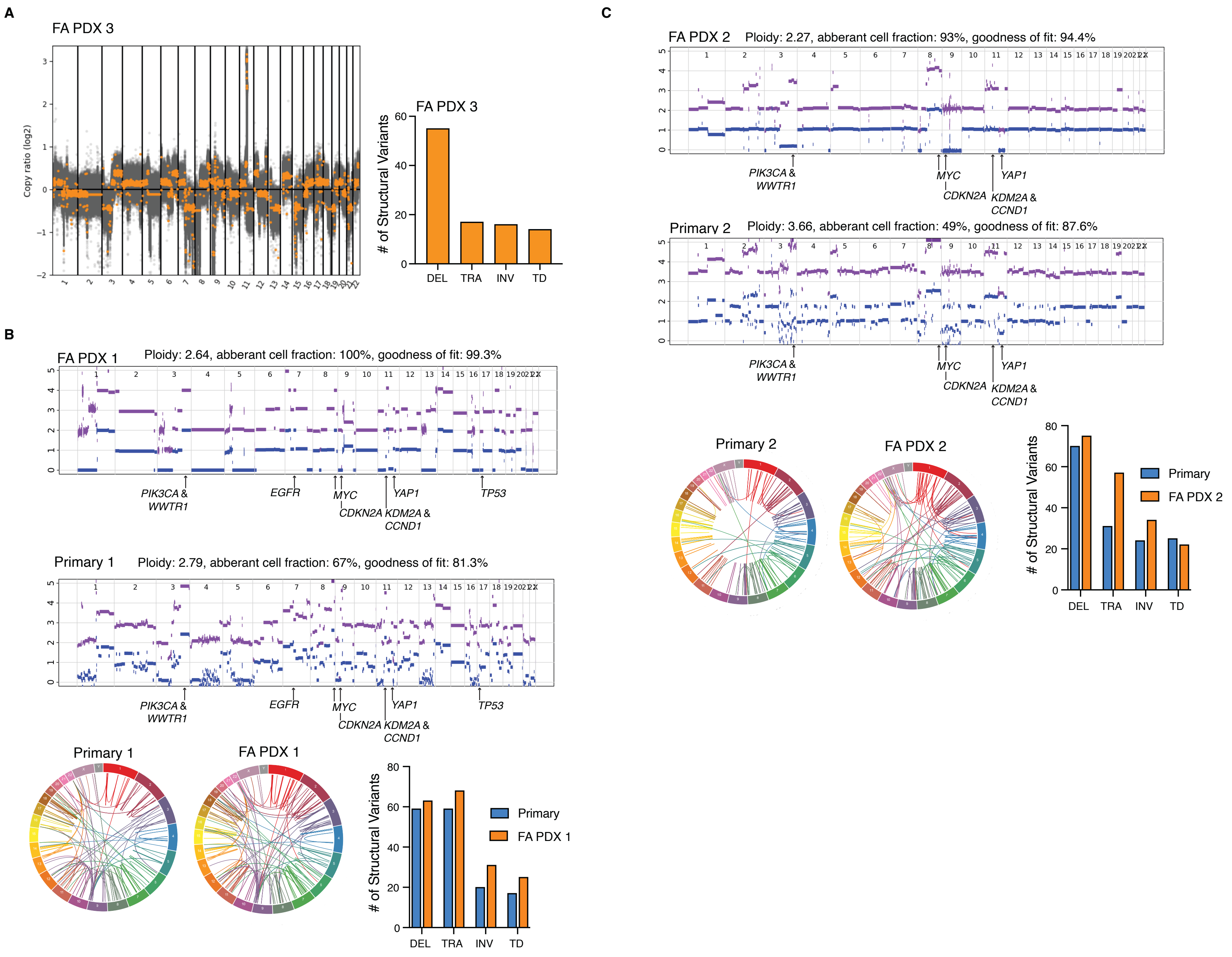

Supplemental Figure 3

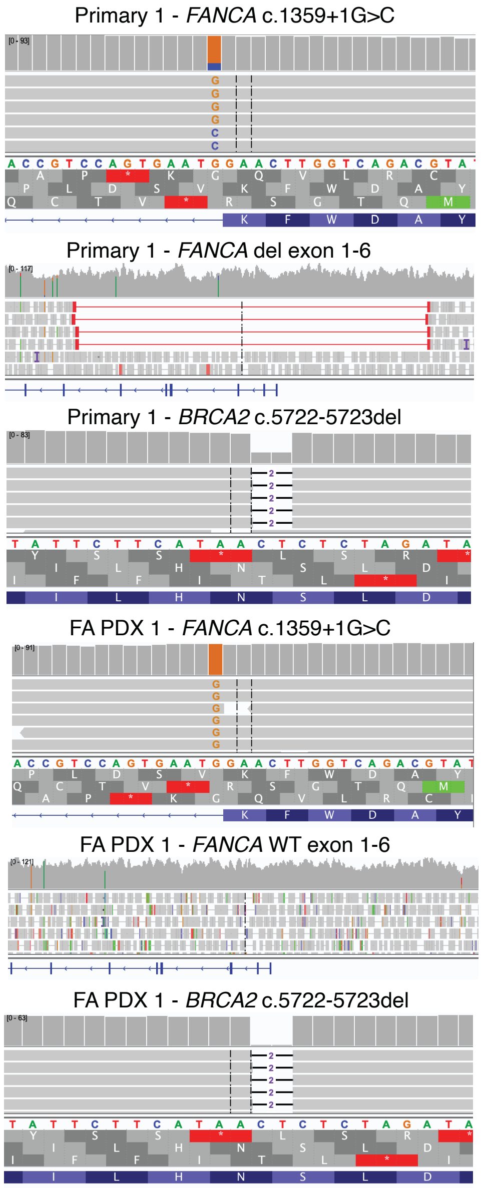

Supplemental Figure 4

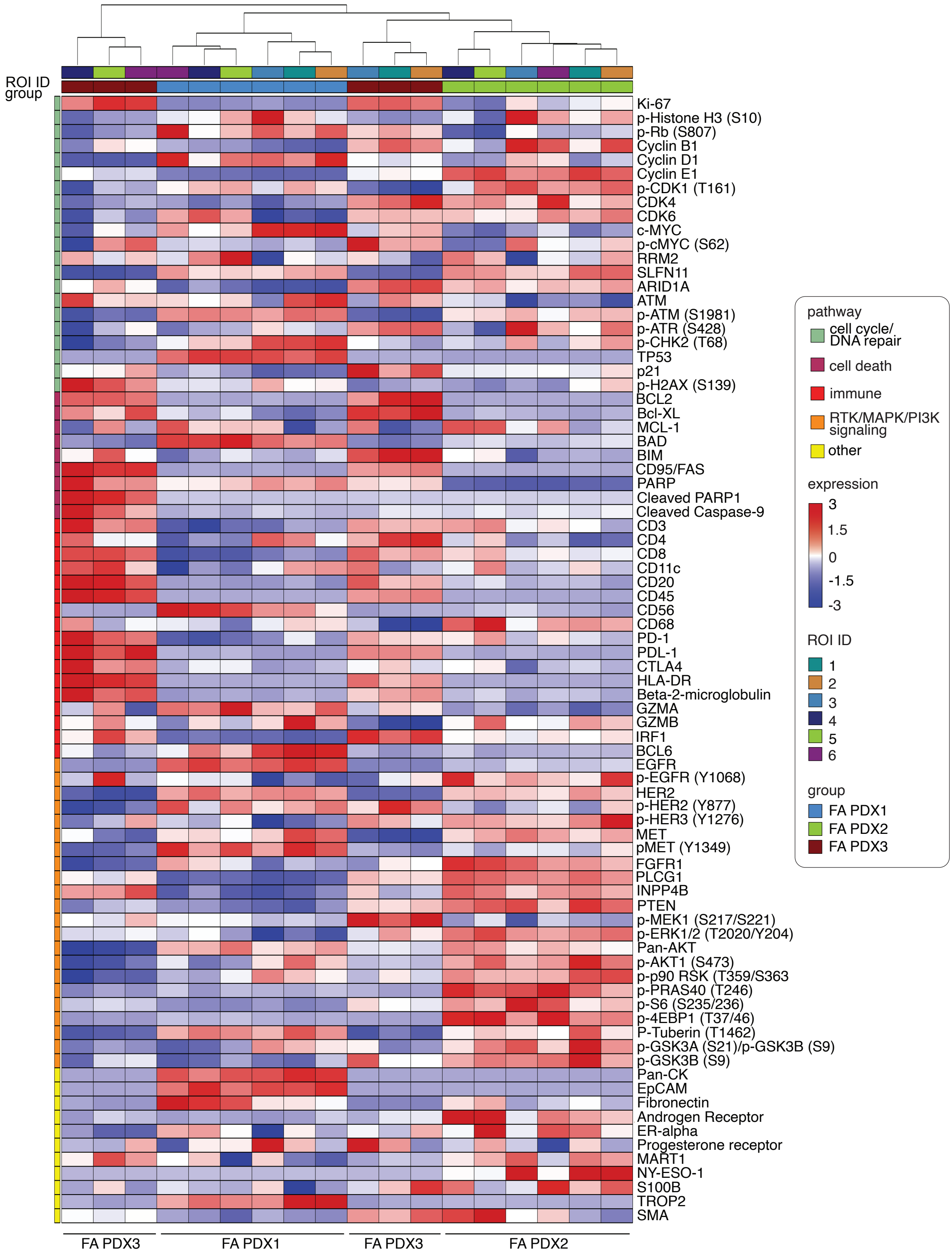

Supplemental Figure 5

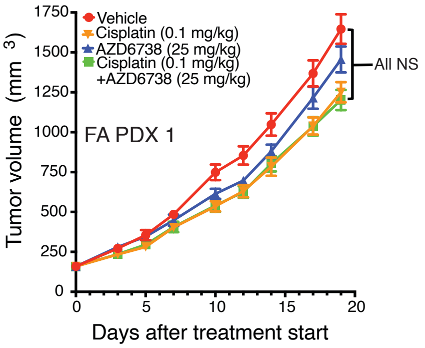

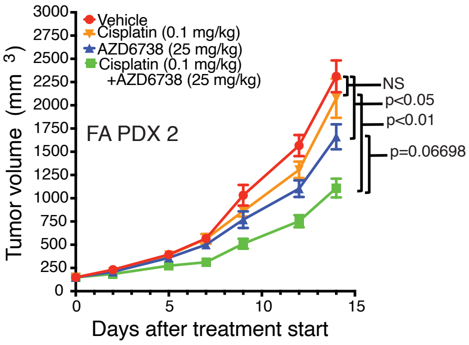

Supplemental Figure 6

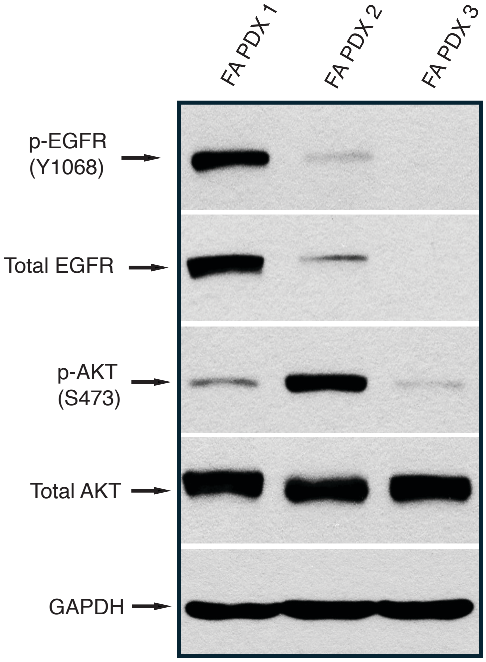

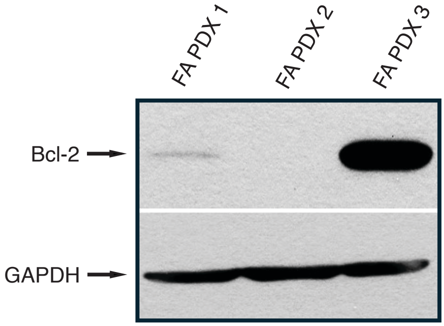

Supplemental Figure 7

FA PDX 3

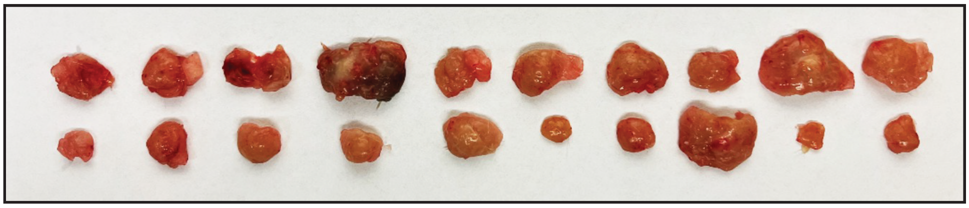

Supplemental Figure Legends and Supplemental Table Legends

Supplemental Figure 1. Hematoxylin/Eosin staining of the FA-HNSCC PDX models

Supplemental Figure 2. Genomic characterization of FA-HNSCC patient-derived xenograft models and their primary counterparts. (**A**) Graph showing copy number across chromosomes as identified by CNV kit for FA PDX 3 (left). Graph with number of somatic structural variants (SVs)s in FA PDX 3 stratified by structural variant class. DEL indicates deletion, TRA indicates translocation, INV indicates inversion, and TD indicates tandem duplication. (**B**) Genomic analysis of FA PDX 1 and the corresponding primary tumor. Top graphs show allele-specific copy number plots of the FA PDX 1 tumor and the primary tumor. Blue and purple lines represent minor and total allele count with y-axes indicating relative copy number across all chromosomes. 1 and 2 are neutral for minor and total alleles respectively. Estimated ploidy, tumor purity, and ASCAT model fit for the FA PDX 1 and primary tumor samples are indicated. Annotated below the graph are key HNSCC oncogenes and tumor suppressor genes with copy-number alterations in each sample. Below are Circos plots displaying somatic SV calls (left) and a graph with number of somatic SVs stratified by structural variant class (right) in the FA PDX 1 tumor and the corresponding primary tumor. (**C**) As in B, but for FA PDX 2 and the corresponding primary tumor.

Supplemental Figure 3. Zygosity status of identified *FANCA* and *BRCA2* mutations in primary and FA PDX 1 tumor samples. Displayed is the loss of the *FANCA* exon 1-6 deletion allele, and complete allelic reversion to *FANCA* c. 1359+1 splice mutation in the FA PDX 1 tumor. Also shown is the loss of the *BRCA2* WT allele and complete allelic reversion to *BRCA2* c.5722-5723 deletion in the FA PDX 1 tumor.

Supplemental Figure 4. Unsupervised heatmap representation of DSP protein targets across samples. Samples are clustered according to Euclidean distance.

Supplemental Figure 5. ATR inhibition modestly enhances sensitivity to cisplatin in FA PDX 2 tumors. Mice harboring FA PDX 1 (6 mice/group and 2 tumors/mouse) or FA PDX 2 (5 mice/group and 2 tumors/mouse) were treated with vehicles (saline once per week by IP injection and/or 0.5% methylcellulose and 0.1% Tween 80, 5 days per week by oral gavage), cisplatin (0.1 mg/kg once per week by IP injection), AZD6738 (25 mg/kg, 5 days per week by oral gavage), or the combination of cisplatin (0.1 mg/kg) and AZD6738 (25 mg/kg). Error bars depict the SEM.

Supplemental Figure 6. Expression of p-EGFR, total EGFR, p-AKT, and Bcl-2 biomarkers in late passage FA PDX tumors. Lysates were prepared from late passage FA PDX 1 (passage 16), FA PDX 2 (passage 28), and FA PDX 3 (passage 23) tumors and subjected to immunoblotting for p-EGFR, total EGFR, p-AKT, total AKT, Bcl-2, or GAPDH.

Supplemental Figure 7. Inhibition of FA PDX 3 tumor growth with venetoclax. Mice with FA PDX 3 tumors were treated 5 times/week with vehicle (0.5% methycellulose + 0.1% Tween 80) or venetoclax (100 mg/kg) via oral gavage (5 mice/group, 2 tumors/mouse) for 18 days. Upon completion of treatment with vehicle or venetoclax, tumors were harvested and photographed.

Supplemental Table 1. Additional clinical history and pathology data from patients whose tumors were used to derive the FA PDX models used in this study.

Supplemental Table 2. Genomic analysis for genes of interest found in HNSCC. Tabs are stratified by sample and type of genomic alteration, including SNVs, indels, and CNVs. CNVs are further stratified by amplifications, deletions, and copy-neutral losses of heterozygosity (cnLOHs). For SNVs and indels, columns indicate insult type. TRUE means that the alteration was found in that gene in the given sample.

Supplemental Table 3. Complete processed genomics files for all samples stratified by SNVs, indels, CNVs, and SVs. These data contain all identified variants of the given type in each sample that were used when analyzing the genes of interest to generate Supplemental Table 2.

Supplemental Clinical information:

FA PDX 1 was generated from a hypopharyngeal tumor in a 13-year-old male with Fanconi anemia. Fanconi anemia was diagnosed at 8.6 years of age after the patient presented with a myelodysplastic syndrome. Germline *FANCA* gene variants were c.1359+1G>C and c.-42-?_596+?del. Shortly after diagnosis, he was conditioned with busalfan, cyclophosphamide, and fludarabine, and underwent an allogeneic stem cell transplant from an HLA identical relative. At 13 years of age, he developed squamous cell carcinoma of the hypopharynx and underwent a biopsy with a bilateral neck dissection, which revealed a moderately differentiated squamous cell carcinoma in a background of squamous cell carcinoma in-situ. All of the 60 lymph nodes resected were free of tumor. Subsequent laryngopharyngectomy revealed a tumor of 3.8 cm in greatest dimension for a final staging of T2N0M0 (Stage II). A piece of the tumor from that resection seeded the PDX. Cancer recurred three months later in the neck with lung metastases at both regional and distant sites of neck and lungs. The patient was given palliative care (no chemotherapy or radiation) and died five months later due to progressive disease.

FA PDX 2 was generated from an oral cavity tumor of a recurrent squamous cell carcinoma in a 38-year-old male with Fanconi anemia. He was diagnosed with Fanconi anemia at age of five using a DNA breakage test and was found to have two variants in *FANCA* (c.1827-1G>A and c.2602-13C>G). Fibroblasts derived from this patient exhibited a lack of FANCA protein expression and no FANCD2 monoubiquitination, consistent with FANCA loss. The patient received a bone marrow transplant from an

HLA-identical nonrelated donor at the age of 13. At 29 years of age, a lower lip cancer was excised, but the pathology report was not available. The lesion recurred at 35 years of age as an ulcerated, moderately differentiated squamous cell carcinoma "of skin of lip" and was excised. Carcinoma extended to submucosal skeletal muscle with depth of invasion of 2 mm. Margins were clear. No adjuvant therapy (chemotherapy and/or radiation) was administered. At 38.4 years of age, a biopsy of the left midline lower gingiva revealed keratinizing squamous cell carcinoma. The patient underwent left marginal mandibulectomy and left neck dissection. The tumor was 3.6 x 2.3 x 1.5 cm in size with a moderately differentiated squamous cell carcinoma histology. Left sided lymph node dissection revealed 22 regional nodes that were all free of tumor (staged pT4a pN0). He was treated with adjuvant radiation therapy to the primary tumor site with radiation starting 47 days post resection and administered over 43 days with total dose of 60 Gy in 30 fractions of 2 Gy. The tumor recurred 6.6 months after original resection (3.5 months after completing radiation) with biopsy revealing malignant spindle cell tumor. TP53 expression was patchy in the tumor, Ki67 expression was 70%. 50% of tumor cells and 1% of immune cells were positive for PD-L1 for a combined positive score of 51.

The patient was treated for two cycles of with a combination of pembrolizumab (200 mg) on day one of the 21-day cycle and paclitaxel (80 mg/m^2 on day 1 and 8 of 21-day cycle) with clinical response. Pembrolizumab was discontinued due to soft tissue inflammation and worsening of osteoradionecrosis in the jaw and immune-related pneumonitis. After a hiatus of three weeks, treatment was continued with weekly paclitaxel (80 mg/m^2) and cetuximab (250 mg/m^2). Paclitaxel was held after the first

dose due to progression of necrosis but cetuximab was continued weekly with total of 4 weekly doses given. 5-FU (500 mg/m^2) and leucovorin (20 mg/m^2) were added approximately 6 weeks after paclitaxel was stopped and were continued together with cetuximab for 5 more weeks. However, the tumor progressed through this treatment. Salvage resection of the midline lower lip, floor of mouth, tongue, mandible, and skin was performed at age 39.5 years, a year from the original gingival SCC diagnosis. From this resection, a sample from the floor of mouth and tongue root was used to generate FA PDX 2. The tumor from this resection measured 9 cm in the largest dimension and had spindle cell variant differentiation on histology. One lymph node was positive for tumor cells. The patient succumbed to disease two months after this resection.

FA PDX 3 was derived from a tongue of a 27-year-old Fanconi anemia patient who was diagnosed with FA at age 6.8 and found to have two germline pathogenic variants in *FANCA* (c.3788_3790delTCT and c.2015-1G>T). At 9 years old, he received a bone marrow transplant from an HLA-identical relative. At the age of 26, the patient was found to have severe dysplasia of the left lateral tongue and inferior upper lateral tongue and underwent surgical excision of this area. Pathology of the surgical sample showed no dysplasia at the resection margins. One year later, biopsy of the left lateral tongue identified an invasive, moderately differentiated, p16-negative, squamous cell carcinoma. The patient underwent left partial glossectomy and bilateral neck dissection. All 23 lymph nodes from both sides were negative for disease (T2N0M0; Stage II). Samples derived from the tongue of this surgery were used to generate FA PDX 3. Nine months after the left tongue resection, the patient underwent fine needle aspiration

of a mass in the right neck which was positive for keratinizing squamous cell carcinoma. No other primary tumor was identified, and the right neck metastasis was presumed to be a contralateral metastasis from the original left tongue tumor. A salvage right modified radical neck dissection was performed, with the 2.2 cm soft-tissue metastatic deposit completely replacing a cervical lymph node. Nine other lymph nodes were found to be negative for disease. Tumor tissue was p16-negative, and PDL-1-positive by immuoperoxidase staining, chromogenic in-situ hybridization for high-risk HPV was negative, targeted mutation analysis for EGFR was negative. The patient was treated with radiotherapy to the neck and is alive 36 months after the tongue resection that was used for PDX generation. Radiotherapy therapy was intensity-modulated image-guided proton radiotherapy using a pencil beam scanning with an expanding field technique, initially targeting the planned target high risk volume to assess radio-sensitivity/response before expanding to larger target volumes. There was no adaptive replanning. Total cumulative dose to high risk planned target volume (HR PTV) was 66 Gy (relative biological effectiveness [RBE] in 33 fractions once daily). The escalation was: 3 days of HR PTV = 3 mm + HR clinical target volume (CTV) (right level Ib - region of neck metastasis) 2 Gy/fraction, 3 fractions, 1 fraction per day; followed by 5 days of intermediate risk planned target volume (IR-PTV) = 3 mm + IR CTV (right level Ib, II, III) 2 Gy/fraction, 5 fractions, 1 fraction per day; followed by 25 days of standard risk planned target volume (SR PTV) = 3 mm + SR CTV (right neck) 2 Gy/fraction, 25 fractions, 1 fraction per day. During therapy, patient developed the expected mild-moderate treatment associates adverse events including Grade I mucositis, Grade 2

dermatitis, Grade 1 xerostomia. He did not require opioid pain medicine, a feeding tube, routine IV hydration, or steroids.
